# Addressing the Societal Challenges in Organizations: The Conceptualization of Mindfulness Capability for Social Justice

**DOI:** 10.1007/s10551-023-05357-5

**Published:** 2023-02-21

**Authors:** Yanina Rashkova, Ludovica Moi, Francesca Cabiddu

**Affiliations:** Department of Economics and Business, University of Cagliari (Italy), Viale Sant’Ignazio 74, 09123 Cagliari, Italy

**Keywords:** Mindfulness, Social justice, Organizational capability, Habitual organizing, Sustainable organizational development

## Abstract

Social inequalities are partly caused by habitual organizational practices. In this vein, to overcome those, organizations now need to develop new organizational capabilities aimed at enhancing their attention towards societal issues. In our study, we apply the theory of mindfulness to explain how it may help organizations overcome habitual organizing that fuels social inequalities. Guided by the microfoundational perspective of organizational capability, we conceptualize individual characteristics, processes, and structures that collectively form mindfulness capability for social justice. We perceive it as an organizational capability that reflects the extent to which an organization possesses a collective social justice awareness, i.e., awareness of the impact on social justice in society through its organizational practices. We argue that, when adopted by organizations, mindfulness, by increasing the awareness of the organizational impact on society, helps notice, examine, and question the correctness of taken-for-granted organizational practices. From our perspective, this new capability will lead to changes in organizational practices that fuel social inequalities. Our study contributes to the literature on sustainable organizational development and mindfulness research in organizations. Managerial implications and future research directions are also discussed.

## Introduction

Social justice, a state where human rights and resources are distributed fairly, moderately, and equitably in society (United Nations, 2006), represents a fundamental step toward a prosperous human future (Sustainable Development Agenda, United Nations, 2015). Organizations, regardless of their size and activity sector, being the core of any society, cover a primary role in influencing social justice issues—such as income gap, climate justice, and racial injustice—triggering, with their business practices, a significant social transformation (Bapuji et al., 2020; Sandel, 2020).

From a business perspective, accounting for social justice helps firms enhance their corporate social performance, hence, their social impact and commitment to people within and outside the organization from social, environmental, and economic perspectives (Wood, 1991). Indeed, recognizing the need for more sustainable development, in which social justice is an important component, organizations have been increasingly adopting a triple bottom line (TBL) approach (Elkington, 1998, 2018). For decades, organizations have been incorporating corporate social responsibility practices to redesign organizational practices and account for all kinds of responsibilities related to running a business (economic, legal, ethical, and philanthropic) (Carroll, 1979, 2021). In this vein, extensive research of theories contributed to explaining factors that enhance sustainable organizational development. For instance, the institutional theory posits that organizations would be more likely to act in socially responsible ways if there were strong and well-enforced state regulations in place to ensure such behavior (Campbell, 2007). The resource-advantage (R-A) theory adds that personal moral codes of the owners’ and firms’ executives and consumers’ sustainability orientation, among others, contribute to endorsing more socially justice-oriented values and behaviors (Hunt, 2017).

Although today more and more companies are committed to redesigning their activities to positively contribute to the development of social justice (Harrison et al., 2019; Lazzarini, 2021), several profound economic, political, and environmental injustices are still ingrained in many organizational practices, such as that of hiring, promotion, compensation, role allocation, and structure (Amis et al., 2020). One of the main reasons why organizations continue to reproduce social inequalities lies within their taken-for-granted way of operating (Amis et al., 2017, 2020; Powell & Rerup, 2017). As most practices and processes established in organizations come from repetition, over time, it gives rise to “habitualized actions” (Berger & Luckmann, 1967) that progressively gain legitimacy and become institutionalized throughout the organization. For instance, in recruitment processes, still, many firms use tools that target specific privileged groups or a specific ethnicity, thus contributing to boosting inequality in hiring practices (Amis et al., 2020). Therefore, as long as organizations leverage upon practices and processes that trigger inequality, they create and reproduce social inequalities.

Despite the advancement of previous research on sustainable organizational development (e.g., Campbell, 2007; Carroll, 1979, 2021; Hunt, 2017; Maon et al., 2009), we still lack the understanding of how organizations may overcome non-reflexive and habitual ways of organizing that fuel social inequality. And although routines and habitual behavior are essential for organizational learning and process optimization, not all routines, and practices established in an organization lead to the positive development of social justice in society (Amis et al., 2020). Developing organizational capabilities that will adjust processes and practices according to the impact they have on society represents an important step toward more sustainable organizational development (Bapuji et al., 2020; Sandel, 2020).

To explain how organizations may overcome habitual organizing, we turn to mindfulness theory. Mindfulness is defined as cognitive ability stemming from “social practice that leads the practitioner to an ethically minded awareness, intentionally situated in the here and now” (Nilsson & Kazemi, 2016, p. 190). It provides a solid framework that breaks habitual behavior (Lueke & Gibson, 2014) and brings a deeper understanding of the causes, consequences, and realization of an interdependent form of all well-being (Greenberg & Mitra, 2015; Yu et al., 2020). Notably, it decreases automaticity by reducing the interpretation of events in a typical or routine manner (Brown & Ryan, 2003). As has been frequently suggested by previous research, mindfulness can lead to increased sensitivity and attention to societal issues in organizations (Hick & Furlotte, 2009; Sajjad & Shahbaz, 2020; Wamsler et al., 2018). In this vein, it may help organizations to turn away from past habitual organizing and reflect on how present practices could shape the future.

Despite the initial glance of a positive relationship between individual mindfulness and social justice (Hick & Furlotte, 2009; Rashkova et al., 2022; Schuh et al., 2019) and the recognized application of collective mindfulness (Vogus & Sutcliffe, 2012), the research on mindfulness in organizations is still very limited and not yet applied at a societal level (Qiu & Rooney, 2019; Sajjad & Shahbaz, 2020; Schneider et al., 2010; Wamsler et al., 2018). This inquiry of the research is also in line with the previous literature calling for the examination of justice perception at a collective level in organizations (Schminke et al., 2015). Moreover, despite the extensive contribution of previous theories on building sustainable organizations, it is still unclear how organizations may overcome the non-reflective way of organizing and enhance the awareness of the organizational impact on society (Amis et al., 2020; Bapuji et al., 2020). To address these gaps, we aim to answer the following research question: “How does mindfulness help organizations overcome social inequalities?”

To address our research question from an organizational level perspective, we use a microfoundational lens of organizational capability. Organizational capability is traditionally defined as a unique, aggregate combination of resources targeted to achieve a specific result (Teece, 2007). To explain how mindfulness may help overcome habitual organizational behavior and consequently contribute to sustainable development, we conduct an integrative literature review (Torraco, 2005). We identify three main building blocks or “microfoundations” considered by scholars as the founding base of each capability: individuals, processes (the way people interact), and structures (the way people are organized) (Barney & Felin, 2013; Felin et al., 2012). Our research gives rise to the Mindfulness Capability (MC) for Social Justice, which we perceive as an organizational capability that reflects the extent to which an organization is aware of its impact through organizational practices on social justice in society.

This study has several contributions. First, we contribute to the theories of sustainable organizational development (Campbell, 2007; Carroll, 1979, 2021; Hunt, 2017; Maon et al., 2009; Wood, 1991). By conceptualizing MC for Social Justice, we explain how mindfulness may instill collective awareness of social justice, which is one of the components of sustainable development. We argue that when supported by specific organizational processes and structures, mindfulness help organizations recognize the impact organizational practices have on society and consequently overcome social justice issues embedded in their business practices. As social inequalities are reinforced by past automatic and habitual organizing (Amis et al., 2017, 2020), we explain how mindfulness, by bringing greater awareness to the here and now, helps notice, examine, and break taken-for-granted organizational practices that fuel present and future social inequalities. Therefore, when adjusting or adapting their organizational practices to provide more effective responses to the market changes, organizations that nurture a MC for Social Justice will be more prompt to intervene and correct not responsible habitual organizing and eventually prevent social inequalities.

Moreover, we contribute to the literature on mindfulness in a business context. Notably, by developing a multilevel mindfulness capability framework, we explicate how mindfulness fosters social justice at the individual, organizational and societal levels. Notably, on an individual level, we provide the underlying mechanisms through which mindfulness impacts individuals’ awareness of social justice that leads to more social justice-inclined behavior. On a collective level, by discussing organizational processes and structures contributing to collective social justice awareness, we extend the literature on collective mindfulness by conceptualizing a new organizational mindfulness capability that goes beyond the organizational level, i.e., to the societal level. Moreover, considering both perspectives in this study, we move mindfulness research toward a multilevel mindfulness theory (Sutcliffe et al., 2016). By providing a set of concrete practices, we assist managers looking to redefine their activities to contribute to the development of a just society. We discuss how managers may measure and develop collective social justice awareness and why it is important.

## Theoretical Background

### Sustainable Organizational Development: Social Justice Perspective

Organizations, regardless of size and activity sector, constantly affect multiple stakeholders and influence the development of many social justice issues, such as racial injustice, income gap, and climate justice, which in turn define the social and economic status of the vast majority of people around the world (Bapuji et al., 2020; Sandel, 2020). Indeed, the organizational practice of hiring, promotion, compensation, role allocation, and structure may constantly reproduce social inequalities (Amis et al., 2020). It has been similarly noted the unfairness of work flexibility arrangements between upper and lower-level employees, leaving lower-level employees unable to flexibly control their work (Kossek & Lautsch, 2018). Such inequalities have been even further exacerbated during the Covid-19 crisis (Bapuji et al., 2020).

Recognizing their social justice responsibilities, organizations have begun to adopt a 'triple bottom line' (TBL) approach (Elkington, 1998, 2018), focusing simultaneously on the ecological, social, and economic aspects. They have also started to account for their activities about corporate social responsibility on environmental, social, and governance reports (Sethi et al., 2017). Extensive research has been produced to move toward more sustainable organizational development. Within the institutional theory, previous research highlight that in a highly regulated and stimulating environment, organizations are naturally more likely to behave in a more socially responsible manner (Campbell, 2007; Huq & Stevenson, 2020). Supporting the institutional theory, the R-A theory adds that the personal moral codes of the owners and firms’ executives, together with consumers’ sustainability orientation, among others, contribute to the endorsement of more social justice-oriented values and behaviors (Hunt, 2017).

On a micro-level, undoubtedly, economic conditions influence the behavior of an organization and largely determine the strategic course of social action (Drempetic et al., 2020; Waddock & Graves, 1997). For example, economically viable organizations can allocate resources to support their social programs. Furthermore, internal organizational processes and policies determine how far sustainable organizations are (Shin et al., 2015; Wood, 1991). For instance, organizational culture, management tenure, and financial performance (Melo, 2012) impact the degree to which an organization is attentive to social justice. Similarly, collective perceptions of moral values in organizational settings influence collective perceptions of justice in organizations (Schminke et al., 2015).

The question of why social justice matters to a business can be explained from two main perspectives: business and societal. From a business perspective, incorporating social justice principles into business operations enhances its long-term sustainability, which investors widely recognize as a sign of an organization's ethical bent and corporate goodwill (Bucaro et al., 2020). Accounting for social justice also responds to the ever-increasing expectations of consumers regarding business goals. As more and more consumers call for more inclusive and sustainable business operations that benefit society and the planet (He & Harris, 2020), socially justice-driven practices will positively impact consumer loyalty and retention. From a societal perspective, a socially sustainable business significantly contributes to human development and creates added value for all stakeholders, thus alleviating the social exclusion of people (Bocken et al., 2014).

However, despite the widespread adoption of a 'triple bottom line' (TBL) approach (Elkington, 2018), CSR principles in organizations (Carroll, 2021), pertinent regulation of institutions, and strong incentives for leaders, organizations today continue to fuel many social inequalities (Amis et al., 2020; Bapuji et al., 2020). Part of the answer to why the above factors do not always work lies in habitual and stacked organizational behavior. Since all types of practices and processes established in organizations arise from repetition, these practices become ‘typification of habitualized action’ (Berger & Luckmann, 1967, p. 54). Habitual organizing leads to practices becoming legitimized and institutionalized, allowing social inequalities to be reproduced within organizations (Amis et al., 2017, 2020; Powell & Rerup, 2017). Although routines are essential for organizational learning and process optimization, not all routines established in an organization can lead to the positive development of society (Amis et al., 2020). The ability to adjust habitual processes and practices according to the impact they have on society represents an important step towards a more socially just society. In this regard, we propose to build upon mindfulness theory to help organizations overcome non-reflexive and habitual ways of organizing.

### Mindfulness

In the literature, mindfulness is studied on both collective and individual levels. On an individual level, research into the relationship between individual mindfulness and social justice is still in its infancy (Sajjad & Shahbaz, 2020; Wamsler et al., 2018) and is fragmented across multiple research domains. Within social work studies, mindfulness was proposed as a method to uncover, analyze, and change unjust social structures and institutions (Hick & Furlotte, 2009). In the business domain, previous research has revealed that a leader’s mindfulness is positively related to a leader’s enactment of procedural justice toward employees (Schuh et al., 2019).

On a collective level, mindfulness represents an organizational capability that ensures a firm’s resilience and efficiency in conditions of stress and a highly responsible environment (Vogus & Sutcliffe, 2012). The concept is frequently studied in the context of High-Reliability Organizations, such as, for example, aircraft carriers or nuclear power plants where one error or deviation from the normality may cost human lives (Sutcliffe et al., 2016; Vogus & Sutcliffe, 2012; Weick & Sutcliffe, 2006). Collective mindfulness is built upon a conceptualization of individual mindfulness as a way of processing information (Langer & Moldoveanu, 2000),^1^ where, by increasing the quality of attention, mindfulness improves information processing, which leads to advanced cognitive differentiation and creativity (Hart et al., 2013). Authors argue that to strengthen mindful operating on a collective level, a set of processes and structures should be adopted, namely sensitivity to operations, reluctance to simplify, preoccupation with failure and success, deference to expertise, commitment to resilience (Weick & Sutcliffe, 2006), and the most recent one—comfort with uncertainty and chaos (Fraher et al., 2017). Although important for ensuring constant operation, organizational mindfulness capability for resilience does not account for a collective capability for social justice.

To conceptualize a new mindfulness capability, we build upon a definition that combines both perspectives on individual mindfulness (Western and Eastern) and emphasizes the importance of the socially engaging element of mindfulness that elicits compassion for others and awareness of the possible impact on others (Nilsson & Kazemi, 2016). The definition of mindfulness as “social practice that leads the practitioner to an ethically minded awareness, intentionally situated in the here and now” (Nilsson & Kazemi, 2016, p. 190) is indeed more in line with social justice studies, where scholars support a society-centric definition of mindfulness (Hick & Furlotte, 2009).

Particularly, focusing on the here and now (i.e., the present) can bring valuable potential for organizations in their attempt to bridge social inequalities. Because social inequality stems in part from habitual organization reinforced by past habitual actions (Amis et al., 2017, 2020; Powell & Rerup, 2017), abandoning preconditioned reasoning by focusing on the present can raise awareness of alternative or novel organizational solutions aimed at mitigating social inequality. Moreover, to positively impact society in the future, organizations need to reflect today on the consequences of their ongoing practices. In this vein, turning away from past habitual practices and reflecting on the possible future impact on society in the present moment can bridge social inequalities.

This reasoning is supported by the dual processing model of cognition (Kahneman, 2011; Thomson & Bates, 2022). According to the literature, System 1 is characterized by rapid, automatic thinking in response to stimulus perception. System 2, in contrast, involves slower, more controlled, and analytical engagement (Kahneman & Frederick, 2005). As typically relying on System 1 processing, humans render them vulnerable to stereotypical thinking, reliance on previously established associations, and prejudiced reasoning, which all in all lead to a negative evaluation of minorities (Greenwald & Krieger, 2006).

By decreasing path dependence, i.e. those processes that are ‘unable to shake free of their history’ (David, 2001, p. 19), mindfulness frees people to actively and fully engage with phenomena with less bias from those past associations. Indeed, previous research has shown that mindfulness helps people overcome different forms of automatic processing, such as implicit bias (Lueke & Gibson, 2014, 2016), and correspondence bias (Hopthrow et al., 2017). By breaking automatic tunnel reasoning (Brown & Ryan, 2003), mindfulness induces greater reliance on System 2, or on a more objective and controlled evaluation. Moreover, it gets a deeper understanding of the causes, consequences, and realization of an interdependent form of all well-being (Greenberg & Mitra, 2015; Yu et al., 2020). In this vein, we assume that, when implemented in organizations, it may equally reduce the interpretation of events in a typical or routine manner. In the following sections, we exemplify how mindfulness may lead to the breaking of habitual behavior on a collective level in relationship with social justice.

To better unpack the relationship between mindfulness and social justice and, most importantly, understand how it can help organizations overcome social inequalities stemming from habitual organizing, we apply the organizational capability theory of microfoundations (Felin et al., 2012). This perspective enables us to develop a theory-based, multidimensional model of our focal construct, mindfulness capability for social justice, taking into account not only individuals but also collective activities taking place in an organization (Sutcliffe et al., 2016).

## Research Method

The purpose of our review is to summarize the existing research on mindfulness and its relationship with social justice to explain how it can overcome social inequalities stemming from habitual, not socially responsible organizational practices through the development of a new organizational capability. To achieve this end, we carry out an integrative review, a method of literature review recommended when analyzing diversified knowledge and broad research questions (Snyder, 2019; Torraco, 2005), to synthesize research on a topic in an integrated and novel way (Torraco, 2005).

### Data Collection

Using Scopus and Web of Science databases, we used a Boolean search string to retrieve articles that included the words (e.g., “social” or “societ*” or “ethic*” or “justice” or “collective” or “community” or “moral” or “inequality” AND “mindful*””) in their titles, abstracts, or keywords. To ensure that our analysis would encompass and capture the latest developments and trends in the topic, we collected articles published up to December 2021 and restricted our search to the following research domains: Business, Management, Ethics, and Social Issues. In terms of publication outlets, to identify the articles to be included in our analysis while also ensuring a certain level of academic rigor, we began with the top journals (e.g., Journal of Management, Journal of Business Ethics).

After the duplicates had been removed, the procedure yielded 316 articles. We read the abstract of each paper carefully and screened the articles based on their relevance to the scope of our study (Calabrò et al., 2019). To ensure the inclusion of the most relevant works, we also examined conference proceedings and academic books with a reputation for quality. We did not apply any restrictions in terms of methodology and included both empirical and theoretical studies for a more comprehensive understanding of the phenomenon (Bandara et al., 2015).

We considered as non-relevant those articles that studied mindfulness in medical healthcare or did not examine within the confines of social justice and inequality (e.g., the effect of mindfulness on individual outcomes, such as productivity or creativity). Therefore, through hand searching and citation tracking (Rashman et al., 2009), we included other influential papers not caught by our initial keyword search. Following the above process, the resulting data set results in 59 academic articles and two conference proceedings covering 20 years of academic debate and a broad range of academic fields.

**Fig. 1:**
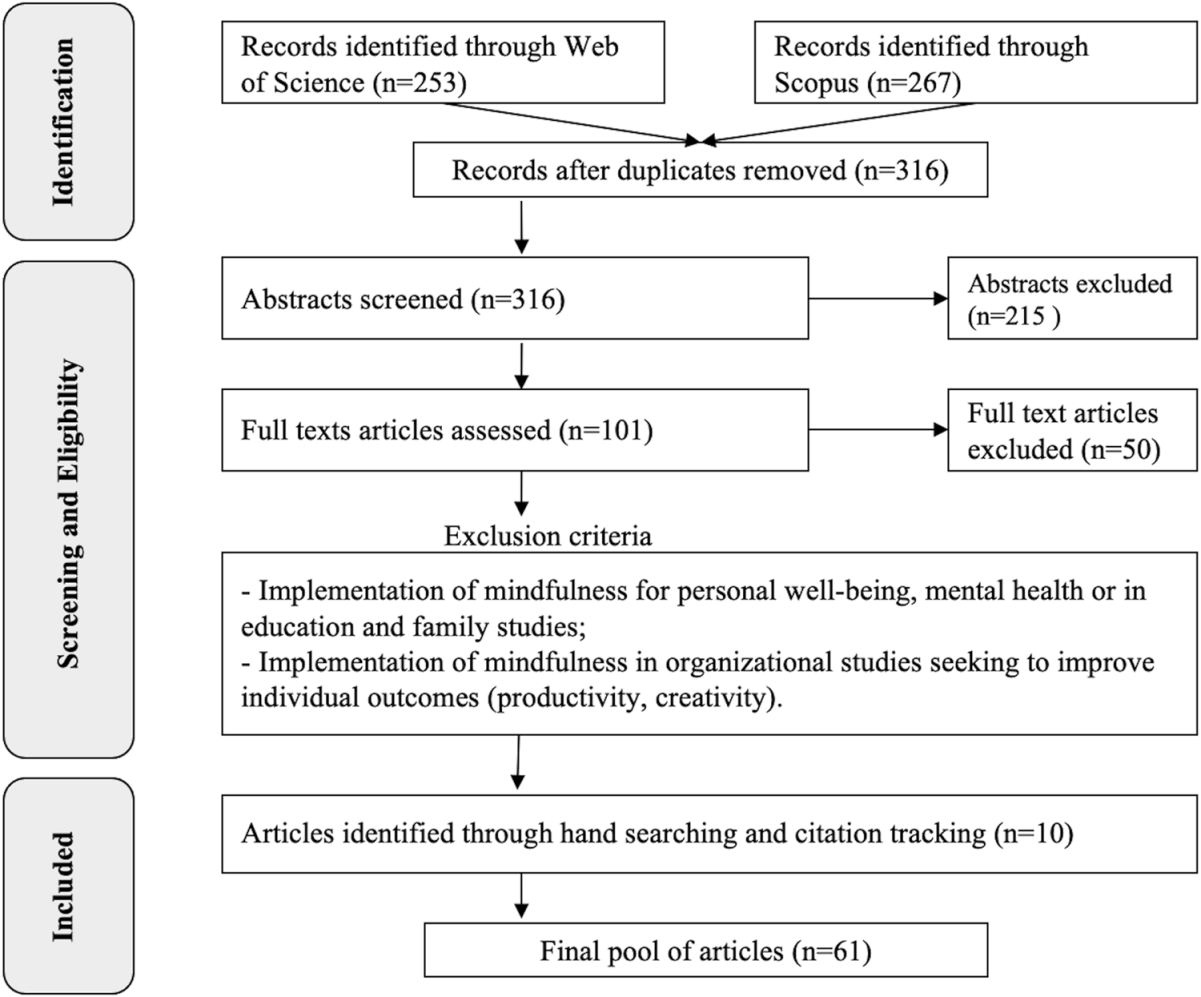
Data collection process. *Source:* own elaboration

We then read and categorized the selected papers based on their relevance to an individual or collective level of analysis. Some papers were included in both phases of the analysis as covering data useful to explain both individual and collective outcomes to social justice. We reviewed 46 articles relevant to the analysis of individual mindfulness to social justice and 23 articles relevant to the collective level of mindfulness to social justice. Figure 1 provides a visual summary of the whole data collection process.

### Data Analysis

To build a multi-level theoretical framework, which encompasses individual and collective constructs, data analysis was carried out in two stages across individual and collective levels of research phenomena. At the individual level, data analysis was guided by deductive reasoning. As such, the codes were defined a priori based on constructs obtained from the previous literature (Miles et al., 2014). This procedure helped to classify mindfulness characteristics and their implications and relationship with social justice.

The second round of data analysis was guided by abductive reasoning (Behfar & Okhuysen, 2018), i.e., the process of reasoning from existing knowledge to the development of novel theoretical concepts. We constantly revised and reassessed identified constructs and relationships between them during the data analysis until we produced a plausible explanation.

Both levels of analysis were performed following the categorization process (Grodal et al., 2020). At first, we approached data by identifying relevant themes via open coding (Gibbs, 2007). Second, we iteratively analyzed data by dropping, merging, splitting, and relating categories till developing robust theoretical constructs. Both coding procedures were implemented in Nvivo 12 software, which can support coding, analyze large amounts of content, link data, display findings, and support theory development (Bazeley & Jackson, 2013). Two authors carried out the analysis process independently and simultaneously and discussed emerging inconsistencies until reaching an agreement.

To integrate the common and complementary elements of theoretical perspectives in the conceptual model, we employed the triangulation of theories method (Denzin, 2017). As guided by the organizational capability theory of microfoundations, we derived a theory-based framework. To simulate the functioning of the framework, we made a collective effort to correlate findings from both sections of our study.

## Findings

**Fig. 2:**
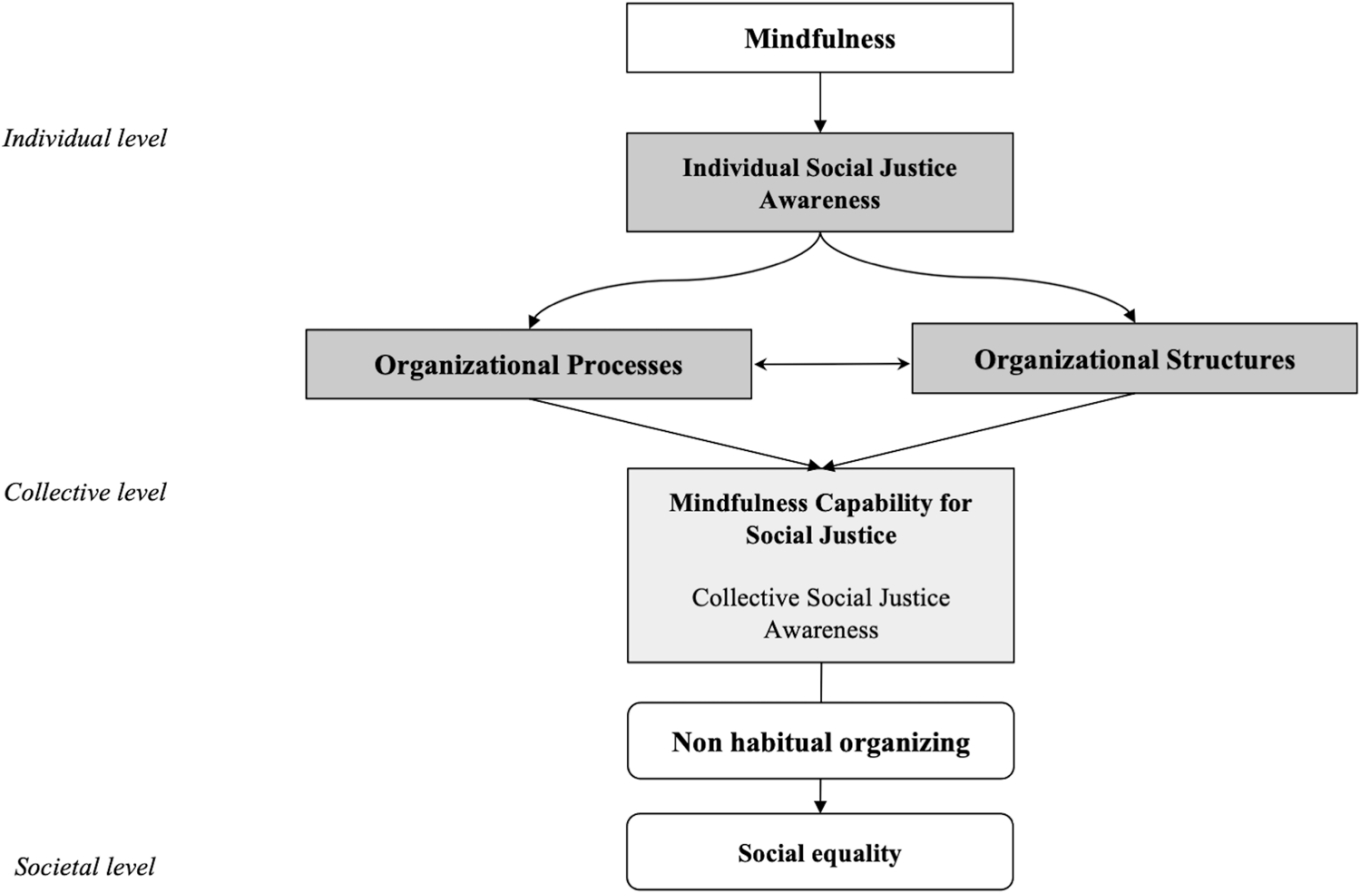
Conceptual framework of Mindfulness Capability for Social Justice: bringing higher social equality at the individual, organizational and societal levels. *Source:* own elaboration

To exemplify how mindfulness may help organizations overcome social inequalities, we first explain the relationship between mindfulness and social justice at an individual level. We show how mindfulness may lead to, what we call, Individual Social Justice Awareness (see Table 1). Second, we identify processes and structures that, in an organizational context, contribute to the development of the collective ability to be more aware of social justice. In closing our paper, we propose a multi-level framework that combines individual and collective perspectives to explain the development of a Mindfulness Capability for Social Justice (Fig. 2).

**Table 1:** Individual social justice awareness: mindfulness mechanisms, implications, and outcomes

Mindfulness mechanisms	Implications	Outcomes
Inner awareness	(−) Cognitive biases (Gill et al., 2020; Lueke & Gibson, 2014; Ransom et al., 2021)	(−) Prejudice and discrimination propensity (Berger et al., 2018; Gervais & Hoffman, 2012; Lueke & Gibson, 2014, 2016)
Outer awareness	(+) Interconnectedness with others (Brown et al., 2007; Pandey et al., 2018) (+) Empathic propensity towards others (Cheung, 2016; Jones et al., 2019; Scherer & Waistell, 2018; Weng et al., 2013)	( +) Social justice attitude and perception (Cartabuke et al., 2019; Hick & Furlotte, 2009; Schuh et al., 2019; Yu et al., 2020) (+) Altruistic orientation and response (Hafenbrack et al., 2020; Iwamoto et al., 2020; Wallmark, et al., 2013; Weng et al., 2013)
Present- oriented awareness	(−) Self-entrainment (Ericson et al., 2014; Kalafatoğlu & Turgut, 2017; Shapiro et al., 2008; Yu et al., 2020) (+) Sense of community (Akin & Akin, 2015; Cheung, 2016)	(+) Prosocial behavior (Cameron & Fredrickson, 2015; Donald et al., 2019; Hafenbrack et al., 2020; Kumar et al., 2020; Weng et al., 2015)
Non-discriminatory awareness	(+) Reconfiguring knowledge (Bahl et al., 2016; Hick & Furlotte, 2009) (+) Objective response (Brown et al., 2007; Forbes, 2016; Rooney et al., 2021)	(+) Recognition of unjust behavior (Burton & Barber, 2019; Culiberg & Mihelič, 2020) (−) Attachment to prejudice via decreased cognitive rigidity (Greenberg et al., 2012; Moore & Malinowski, 2009)
Ethical- minded awareness	(+) Discrimination of whole and unwhole actions (Greenberg & Mitra, 2015; Purser & Milillo, 2015	(+) Moral reasoning (Pandey et al., 2018; Sajjad & Shahbaz, 2020; Shapiro et al., 2012) (+) Ethical behavior and decision-making (Cheung, 2016; Kalafatoğlu & Turgut, 2017; Mihelič & Culiberg, 2019; Orazi et al., 2021; Ruedy & Schweitzer, 2010; Wan et al., 2020)

*Source:* own elaboration

### Individual Social Justice Awareness

Based on the previous literature, our study identifies five mindfulness mechanisms that explain, at an individual level, the relationship between mindfulness and social justice, namely: inner awareness, outer awareness, non-discriminatory awareness, present-oriented awareness, and ethical-minded awareness (see Table 1). According to prior research (Brown et al., 2007; Shapiro et al., 2006), mindfulness characteristics are rarely clearly distinctive since they often overlap and mutually support each other. As a result, in our discussion, mindfulness characteristics are not seen as separate components; instead, they are interpreted as attracting “particles”, which reinforce and nurture each other.

Taken together, these individual-level mindfulness mechanisms lead to Social Justice Awareness. Assuming an ethically-enhanced definition of mindfulness that elicits compassion for others and awareness of the possible impact on others (Nilsson & Kazemi, 2016), we perceive Individual Social Justice Awareness as the individual’s ability to recognize social justice issues and the impact of one’s actions on others in society. By social justice issues here, we mean social problems that impede society from optimal functioning and prosperous development. Examples may include social oppression, income gap, climate justice, and other social inequalities. We also highlight that social justice awareness differs from social awareness, which is the ability to take the perspective of and empathize with others (Wegner & Giuliano, 1982). Through the text, we further exemplify the ways in which identified mindfulness characteristics (see column “implications” of Table 1) impact a range of socially justice related attitudes and behavior (see column “outcomes” of Table 1).

#### Inner Awareness

Prior research unanimously recognizes awareness as a fundamental precondition to achieve a mindful state (Brown et al., 2007; Kabat-Zinn, 2005; Shapiro et al., 2006). Inner awareness is here defined as the quality of paying attention to internal feelings, thoughts, and emotions in a non-judgmental and open way (Brown et al., 2007; Kabat-Zinn, 2005; Nilsson & Kazemi, 2016). Unlike habitual and automatic responses, inner awareness is cultivated by (re)establishing attention toward what one is aware of, without being distracted or mind wandering (Shapiro et al., 2006). In fact, researchers argue that only with focused attention coupled with awareness—acknowledging the current state of the mind that monitors that focused attentiveness—can one become mindful (Good et al., 2016).

Inner awareness supports the development of social justice awareness in important ways. Notably, by paying close attention to the inner state, mindfulness reduces cognitive biases (Gill et al., 2020), i.e., a systematic error occurring through simplified information processing, which leads to decreased prejudice and discrimination among employees. Notably, experimental research has shown that individual mindfulness, induced by mindfulness audio, reduces implicit age and racial biases during implicit association tests (Lueke & Gibson, 2014). Individuals scoring higher in mindfulness are more objective in their judgments toward black and old people, as they do not rely on the automatic activation of negative associations (Langer & Moldoveanu, 2000). In organizations, it may lead to increased inclusion of minority groups into the decision-making process and awareness of, for example, social oppression both within and outside the organizations.

Reduction of cognitive biases also leads to reduction of prejudice and discrimination propensity. Notably, previous research has shown that mindfulness is perceived as an overcoming force that reduces social oppression (Cheung, 2016) and sexism (Gervais & Hoffman, 2012). Moreover, mindfulness leads to reduced prejudice toward outgroups (Berger et al., 2018) and reduced discrimination (Lueke & Gibson, 2016). In organizations, this can translate into employee-increased benevolence towards minority groups, as well as a reduction in negative judgments.

#### Outer Awareness

In our review, we found that outer awareness contributes to forging individuals’ attention to recognize the feelings, thoughts, and needs of others in the environment (Brown et al., 2007; Pandey et al., 2018). Outer awareness evokes a sense of deep connectedness or coexistence with others (Scherer & Waistell, 2018), dissolving boundaries between oneself and other living beings. Cultivating an attitude of universal and unconditional acceptance among employees (Pandey et al., 2018) and moving beyond the egocentric self creates a deep sense of empathy toward others (Cheung, 2016; Jones et al., 2019; Scherer & Waistell, 2018; Weng et al., 2013). An example of outer awareness can be found when leaders understand the motivations of their employees and want to help them to be successful. People with higher levels of interconnectedness shift their focus from the self to the collective and are more willing to endorse social justice ideologies, such as civic engagement, egalitarianism, humanitarianism, and universalism (Yu et al., 2020). Thus, as this sense of interrelatedness and compassion for the world grows, mindful employees may begin to see how their actions influence and perpetuate injustice (Forbes, 2016; Hick & Furlotte, 2009). When mindful, employees and leaders may begin to question the correctness of their taken-for-granted actions and evaluate more profoundly the impact of certain decisions on the broader society.

As inducing empathy (Cheung, 2016; Jones et al., 2019; Scherer & Waistell, 2018; Weng et al., 2013), i.e., the capacity to understand or feel what another person is experiencing, mindfulness significantly relates to social justice attitudes and perceptions. The literature suggests that employees with a higher sense of empathy develop the ability to interactively consider how their actions affect not just their own surroundings but society as a whole (Cartabuke et al., 2019). Moreover, it leads to a greater understanding of the needs of others (Cheung, 2016) and fosters altruistic orientation and response (Iwamoto et al., 2020; Wallmark et al., 2013; Weng et al., 2013). The above reasoning is applicable to organizations where employees are in constant interaction with others. Indeed, as shown by a recent field study, the amount of bonus that participants in the mindfulness condition indicated that they would give to their financially distressed co-workers was greater than the amount indicated by those in the mind-wandering control condition (Hafenbrack et al., 2020).

#### Present-Oriented Awareness

Regardless of the school of thought, previous research on mindfulness argues about the need to return to the state of awareness of what is occurring in the present moment (Nilsson & Kazemi, 2016). It stands to reason that an individual’s mind may tend to "travel in time" between the past and the future (Brown et al., 2007; Kabat-Zinn, 2005. By raising awareness of the present and, therefore, letting the past and future go, present-oriented mindfulness allows people to decentralize from self-entrainment and reduce hedonism (Ericson et al., 2014; Kalafatoğlu & Turgut, 2017; Shapiro et al., 2008; Yu et al., 2020). In an organizational context, mindful employees are more present and attentive toward how the activities are developed and how their actions contribute to the development of social justice.

Being aware of what is currently happening in the environment also enhances empathic participation in social justice issues by creating a stronger sense of community (Akin & Akin, 2015). If employees recognize that the suffering of others can be their own suffering (Cheung, 2016), they are encouraged to participate in solving common problems. In this conception, mindfulness leads to greater social engagement and increased collective empowerment (Cheung, 2016), which is observed in the form of prosocial behavior, both within and outside the organization (Cameron & Fredrickson, 2015; Donald et al., 2019; Hafenbrack et al., 2020; Kumar et al., 2020).

As confirmed by a recent meta-analysis study, people scoring high in mindfulness exercise greater prosocial behavior across age, gender, and helper-recipient relationships (Donald et al., 2019). Moreover, this effect tends to be stronger in people with relatively interdependent self-interpretations (a concept of self that puts oneself in an interpersonal context) compared to independent self-concepts (a concept of oneself as separate from others) (Yu & Zellmer-Bruhn, 2018).

#### Non-discriminatory Awareness

Our review also describes another key mindfulness resource of social justice awareness—non-discriminatory awareness. Non-discriminatory awareness is the individuals’ capacity to observe events without any judgment and discrimination, rather, with an open heart and mind (Brown et al., 2007; Kabat-Zinn, 2005; Nilsson & Kazemi, 2016). In doing so, people do not compare or evaluate events through automatic categorization processes and habitual ways of doing or reasoning (Langer & Moldoveanu, 2000). Indeed, they make assessments based on empirical evidence, such as collecting data on facts occurring here and now (Bahl et al., 2016; Hick & Furlotte, 2009). Such an ability can be useful in reducing unawareness regarding societal issues in organizations (Forbes, 2016). Non-discriminatory awareness induces individuals to analyze the environment without following habitual perceptions or preconceptions and to develop a more objective response (Brown et al., 2007), a less subjective (personal) interpretation of present experiences, and sound judgment (Forbes, 2016; Rooney et al., 2021). This is important, as an abandonment of habitual response in organizations, indeed, provokes the overcoming of social inequalities, as developing the ability to come out from tunnel reasoning and operating.

Non-discriminatory attitude replaces cognitive rigidity (the attachment to old ideals and thoughts) (Greenberg et al., 2012) with cognitive flexibility (Moore & Malinowski, 2009), which provides greater *choicefulness* over whether to allow the automatic responses to run or to consciously regulate behavior in the service of more adaptive outcomes. For instance, when mindful, an employee thinks with great agility and openness and voluntarily considers different points of view during the decision-making process, which allows him to act with greater social justice awareness.

When coupled with ethical-minded awareness, non-discriminatory awareness increases the perception of unjust behavior occurring in the social environment. In this regard, previous empirical research has shown that both dispositional quality and induced state of mindfulness are positively associated with the recognition of unjust behavior. As a result, individuals scoring high in mindfulness were most likely to view abusive behavior as unfair (Burton & Barber, 2019). Similarly, previous research recognizes mindfulness as an antecedent of ethical recognition of peer reporting (Culiberg & Mihelič, 2020). This implies that mindful employees and leaders will be similarly able to pay greater attention to injustices embedded in their organizational practices, which will practically translate into reviewing and establishing organizational practices that do not hinder socially just development.

#### Ethical-Minded Awareness

Finally, individual social justice awareness encompasses ethical-minded awareness, which is related to the moral dimension of people’s everyday actions (Nilsson & Kazemi, 2016; Purser & Milillo, 2015). Similarly, it can be defined as individual wisdom based on an intellectual understanding of the environment (Rooney et al., 2021; Vu & Gill, 2018).

As Buddhist-inspired scholars argue, mindfulness represents an internal guide that allows a person to distinguish between whole and unwhole actions, i.e., between good and bad, on personal and interpersonal scales (Purser & Milillo, 2015). This ability to recognize what is good and what is wrong translates into the ability to recognize what is socially just and what is not in organizations. It may lead to, for instance, the reconsideration of compensation practices and equaling salaries for male and female workers. Therefore, mindfulness can be seen as a transformational tool of attitude, reasoning, and behavior in organizations. The presence of an ethical foundation is necessary for practicing mindfulness since the pursuit of liberation from suffering, when value-neutral, is not necessarily beneficial for society. Notably, previous research recognizes that both dispositional quality (a trait) (Pandey et al., 2018), as well as induction of mindfulness via meditation are associated with improvement in moral reasoning (Pandey et al., 2018; Shapiro et al., 2012).

By decreasing moral disengagement (Brendel & Hankerson, 2021; Wan et al., 2020), mindfulness induces greater ethical behavior, among which less cheating (Ruedy & Schweitzer, 2010), less destructive deviant employees behavior (Wan et al., 2020), reduced ostracism (Christensen-Salem et al., 2021), social loafing (Mihelič & Culiberg, 2019) and increased intention to behave ethically (Kalafatoğlu & Turgut, 2017). Moreover, state mindfulness induced through brief mindful meditation exercises positively causes other-focused ethical behaviors, such as the choice of fair-trade products, charitable giving, and volunteering (Orazi et al., 2021).

## Organizational Processes and Structures of Mindfulness Capability for Social Justice

In the previous sections, our research has shown that, at the individual level, mindfulness leads to cultivating Social Justice Awareness among employees, which is translated into greater awareness of the organizational impact and endorsement of socially just attitudes and behaviors. Nevertheless, at the organizational level, mindfulness cannot be narrowed to single individuals that, for instance, behave mindfully or engage in meditative practices (Weick & Sutcliffe, 2006); rather, it should be nurtured as a dynamic process that involves people and situations (Fraher et al., 2017; Sajjad & Shahbaz, 2020).

To better explain how individual social justice awareness can be extended to the collective level, we apply the organizational capability theory of microfoundations (Felin et al., 2012). This perspective enables us to develop a theory-based, multidimensional model of a new collective ability, considering collective activities in an organization (Sutcliffe et al., 2016). According to the theory, in addition to individuals, which are the most important block, organizational processes and structures are necessary to explain the creation of collective abilities. In this regard, processes are related to how individuals interact with each other, whereas structures are how people are directed and organized (Felin et al., 2012). Therefore, in the next sections, we identify the main organizational processes and structures that organizations should develop to enhance social justice awareness on a collective level.

### Organizational Processes

#### Developing Ethical Practices for Social Justice

The implementation of ethical practices by organizations comes about when they are driven by ethical considerations in several business activities, like production, distribution, and after-sales service (Qiu & Rooney, 2019). For instance, they adopt formal procedures, such as a code of ethics, standards, policies, programs, or more informal methods, as unwritten patterns accepted in groups (Verhezen, 2010). Embedding ethical organizational practices for social justice can reinforce individual social justice awareness and ethical behavior in the whole organization (Beeri et al., 2013; Schwartz, 2001). For example, managers who adopt ethical practices for social justice are guided in solving social justice problems, making decisions, and correctly separating what is right and what is wrong (Beeri et al., 2013).

Based on the prior literature that positively links the adoption of ethical systems and collective mindfulness for sustainability (Nguyen et al., 2020; Umar & Chunwe, 2019; Valentine et al., 2010), we assume that certain practices would increase collective awareness of social justice and break habitual organizing fueling social inequalities. For instance, pursuing a code of conduct or ethics of social justice, accounting for the impact of social justice in decision-making, linking senior management compensation to the achievement of social justice goals, and creating a committee at the board level whose mandate will include monitoring the activities through the societal lenses, altogether, can intensify the collective efforts necessary to enhance social justice in organizations’ performance, operations, and stakeholder relationships.

A practical example of these practices can be found in organizations such as Apple that employ impartial recruitment practices based on the conscious use of recruitment tools that do not jeopardize the chance of being considered for a minority candidate. Similarly, Tesla and Microsoft, to name just a few, hold senior managers and executives accountable for progress on representation and inclusion (Microsoft, 2021; Tesla, 2021).

#### Adopting Social Justice Values

Another process that organizations need to raise the collective rationale and motivation for a commitment to social justice is about the adoption of ethical values, especially those aimed at social justice, to inform mission statements, service provision, and internal organizational culture (Lazzarini, 2021; Sajjad & Shahbaz, 2020). In fact, ethical values at the collective level lead to higher levels of ethical behavior among individuals within the organization through their impact on increased perceptions of distributive and procedural justice (Baker et al., 2006).

Values and virtues can be incorporated into a code of ethics and through the development of an ethical climate (see relevant subclauses) or similarly conveyed by mindfulness teachers or (and) leaders, supervisors, or (and) colleagues who “sow” social justice values within the company (Greenberg & Mitra, 2015). Ingraining the values of social justice into an organizational endeavor, i.e., into organizational culture, service provision, and stakeholder relationships, can therefore become part of its DNA (Verhezen, 2010), which will convey organizational predisposition to the plans and strategies that are more socially just.

For instance, the adoption of a code of conduct inspired by ethical principles that aim to increase societal development is an increasing practice among organizations such as McKinsey, Google, and Apple. Notably, within McKinsey, the commitment to human rights informs not only employees but also clients, which helps them to be mindful of their social impact (McKinsey, 2021). Indeed, the alignment of mindful employees and the adoption of societal values lead to a greater organizational commitment toward social responsibility and the mission of inclusion (Gates et al., 2021). Thus, organizations “imbued” with social justice values​​ increase the level of collective participation in social justice issues, which will ultimately lead to a positive organizational impact on society.

#### Encouraging Interconnectedness

Organizations that foster close collaboration and high employee engagement in the organization are able to create a sense of interconnectedness at various levels. A sense of interconnectedness or perception of close integration with the organizational environment, values, and practices can result from the adoption of various management practices, such as outside workgroup activities, cross-departmental training, and inclusive decision-making.

An example of the desired interconnectedness to be achieved can be described as a family relationship where employees are fully identified with and connected to an organization (Mintzberg, 2009). Processes encouraging employees to actively participate in the organizational conversation lead to greater engagement and commitment to the organization’s goals and day-to-day activities (Ndubisi & Al-Shuridah, 2019). Such commitment, when coupled with the integration of ethical practices for social justice, can increase the opportunity to develop greater social justice awareness on a collective level (Gates et al., 2021).

Moreover, tight relationships with others foster cooperation and trust, which eventually lead to organizational behavior critical for operating in a sustainable way (Dayan et al., 2019). Similarly, positive employee relations have been shown to induce collective mindfulness (Reina & Kudesia, 2020), as they increase sensitivity to operation and commitment toward common goals.

Interconnectedness eventually creates a sense of community (Akin & Akin, 2015; Scherer & Waistell, 2018), which is the feeling of being dedicated to and caring about work, colleagues, and the world around. This makes employees understand how the success of an organization depends on constructive interaction with the communities around it (Yu et al., 2020). Conversely, employees of a company that does not encourage a sense of community can hardly be expected to care about others (Mintzberg, 2009).

For example, volunteering is a part of social agendas in many organizations, such as Clif Corps, where employees are encouraged to dedicate their time to social issues and not-for-profit organizations that are important to them during their working hours (Clif Corps, 2021). Similarly, the creation of networking opportunities outside “preestablished” elite group of potential employees will lead to greater social inclusion. As such, developing and promoting interconnectedness in the workplace can enhance a sense of community, which will incline teams and organizations to be more aware of and inclined to have more socially inclusive goals and practices.

#### Encouraging Diverse Perspectives

Encouraging employees to share knowledge and experience, establishing a decision-making process that takes into account the opinions of several people, and practicing active listening, contribute to establishing diverse perspectives in organizations. The previous research on collective mindfulness for resilience highlights the importance of genuinely encouraging employees to participate in the exchange of ideas and views and to incorporate multiple perspectives for developing mindfulness at a collective level (Fraher et al., 2017; Renecle et al., 2020). In this vein, action learning leadership development programs, i.e., working on problems by gaining new insights in a supportive and confrontational environment of one’s peers, have proved to foster greater attention and mindfulness in teams over the years (Baron, 2016). Furthermore, organizations in which employees freely discuss and question the opinions of others create a sense of security and confidence. A practical example of this can be the organizational process of Tesla, where employees can report concerns to their supervisor or HR partner by anonymously accessing the Integrity Line, available 24 h a day, 7 days a week (Tesla, 2021).

As highlighted by the previous research, organizations that use tools (e.g., digital whiteboards) facilitating the easy exchange of information increase collective awareness across teams (Curtis et al., 2017). Similarly, the introduction of training to overcome unconscious bias and courses led by experts in race and justice, such as at Apple, increase the diversity in organizations that are transmitted in social action (Apple, 2021). Moreover, the practice of building communities within workplace minority groups such as blacks or LGBTQ people, often practiced in many corporations, raises awareness of the needs of underrepresented people.

In summary, broadening perspectives leads to critical analysis in day-to-day operations, resulting in resilient and error-free performance. Likewise, it can broaden horizons during strategy or decision-making, thereby addressing broader issues related to organizational impacts, such as social justice.

### Organizational Structures

#### Non-discriminatory Organizational Climate

Previous research has shown that working climate significantly affects employee mindfulness (Irving et al., 2014; Kalafatoğlu & Turgut, 2019; Lawrie et al., 2018; Reb et al., 2015; Wan et al., 2020). For instance, a caring climate can be perceived as an antecedent of mindfulness in organizations (Kalafatoğlu & Turgut, 2019), while a supportive climate enhances the employee’s mindfulness and organizational citizenship behaviors (Reb et al., 2015). Furthermore, the psychosocial safety climate is an important moderator in the relationship between job control and collective mindfulness (Lawrie et al., 2018).

Similarly, an environment in which participants are attentive and genuinely care for each other positively moderates the relationship between collective mindfulness and ostracism (Christensen-Salem et al., 2021). Thus, organizations that create a sense of active involvement and compassionate interaction with all members will have a greater collective mindfulness effect on ostracism. Likewise, research has shown that the ethical climate in hospitality organizations promotes a more compassionate approach to peers and, therefore, others (Zoghbi-Manrique-de-Lara & Guerra-Baez, 2016).

For example, features of this culture are present in Twitter, where a number of programs have been implemented aimed at creating psychological safety at work. Notably, the company dedicates resources to ensure that every employee has the opportunity to receive counseling and support to improve their mental health (Twitter, 2021).

Perceptions of goodwill, involvement, and caring in teams and organizations, which practically leads to good behavior towards each other, transparent decision-making, and equal treatment of all, can lead to increased recognition of the importance of fair treatment and good practice outside the organization (Reb et al., 2015; Wan et al., 2020).

#### Authentic and Ethical Leadership

Previous literature recognizes that the leadership style is fundamental for the design and creation of organizational activities and processes (Felin et al., 2012; Reb et al., 2015; Sutcliffe et al., 2016) to enhance employees’ mindfulness. Leaders can act as multipliers of collective mindfulness in organizations because of the influence they may exercise on the behavior of many people within organizations (Schuh et al., 2019).

In particular, our review has shown that authentic and ethical leadership has a large impact on the development of a collective inclination toward social justice. It has been found that authentic leadership, i.e., when one preaches what one is doing, plays an important role in developing collective awareness and collective thriving, which ultimately leads to prosocial behavior in organizations (Wu & Chen, 2019). Similarly, ethical leadership was found to be positively linked to compassion at work and interpersonal citizenship behavior, such as, for example, helping out a colleague spontaneously (Zoghbi-Manrique-de-Lara & Viera-Armas, 2019). By merging authentic and ethical leadership (Brown & Treviño, 2006), we can say that leaders who are concerned about others and make ethical decision-making may inspire their colleagues to become sensitized to others’ social exclusion and take action in the form of prosocial justice behavior (Zoghbi-Manrique-de-Lara & Viera-Armas, 2019).

Moreover, the way leaders perceive and experience mindfulness in their personal life significantly influences the way they develop and introduce mindfulness in organizations (Vu & Gill, 2018). As such, leaders that are sensible and aware of social justice issues shape organizations through adopted initiatives and mindfulness practices that foster greater collective attention toward others outside an organization (Christensen-Salem et al., 2021).

## Overcoming Social Inequalities: Mindfulness Capability for Social Justice

Based on our review above, we now propose a framework (see Fig. 2) that summarizes how mindfulness, when implemented in organizations, helps organizations overcome social inequalities and, as a result, fosters social justice awareness at the individual, organizational and societal levels. As our research has shown, on an individual level, mindfulness leads to social justice awareness, perceived as an individual’s cognitive ability to recognize social justice issues and the impact of one’s actions on others in society. When mindful, one does not compare or evaluate the events through automatic categorization processes and habitual ways of doing or reasoning. By increasing attention to social justice issues, individuals within organizations are more inclined to behave in a way to overcome social inequalities embedded in organizational practices.

Although important, individuals alone cannot guarantee the development of collective social justice awareness. However, as individuals represent the primary building block of any organizational capability (Felin et al., 2012), we argue that this is an indispensable basis that gives rise to further collective actions. In this vein, we propose organizational processes and structures that reinforce the development of greater social justice awareness in organizations. Notably, four processes (developing ethical practices for social justice, adopting social justice values, encouraging interconnectedness, and encouraging diverse perspectives and inclusivity) and two structures (non-discriminatory organizational climate and authentic and ethical leadership) lead to the formation of a new organizational capability, *the Mindfulness Capability for Social Justice*. We perceive it as an organizational capability that reflects the extent to which an organization is aware of its impact through organizational practices on social justice in society. Shortly, it represents collective social justice awareness.

As developed at a collective level, some organizational effort is needed to implement and establish the identified processes and structures. Therefore, we assume that the more organizational processes and structures are implemented, the higher the likelihood organizations will develop a Mindfulness Capability for Social Justice. Moreover, we highlight that the identified organizational processes and structures impact each other and ultimately reinforce the development of organizational capability. For instance, ethical leadership is likely to create a working climate where employees are well-treated and cared for, i.e., a non-discriminatory organizational climate.

Once developed in organizations, it will increase awareness of the context, organizational practices, and involved stakeholders and help companies recognize social inequalities embedded in their practices. This increased awareness will help to overcome automaticity and organizational routines that have been drawn on previous practices fueling social inequalities. As mindfulness implies questioning and not being guided by the habitual response, overcoming taking for granted, automated practices will be easier for organizations with developed Mindfulness Capability for Social Justice. We argue that once implemented in organizations, the new organizational capability will impact business practices and operations, which will therefore change corporate behavior toward a more sustainable and socially responsible one. Drawing on the previous literature on sustainable development and, in particular, Corporate Social Responsibility (Maon et al., 2009), where raising CSR awareness inside the organization gives rise to implementing socially responsible practices, we assume that collectively responsible behavior will be preferable in organizations with a developed collective social justice awareness.

It is fair to assume that the higher collective social justice awareness is, the higher will be the probability of overcoming social justice issues embedded in organizations. On the contrary, organizations that lack social justice awareness will be less likely to take responsible action as neglecting and/or not perceiving the interconnectedness of organizational actions with the well-being of society. In this regard, collective mindfulness, as essentially representing the awareness (which comes before any actions) (Maon et al., 2009), represents an initial basis, building impulse that gives rise to further organizational responsible and sustainable actions.

In this first attempt to conceptualize Mindfulness Capability for Social Justice, we argue that its hallmark is its focus on the awareness of organizational practices’ impact as concerns social justice issues within and outside the organization. Although organizations constantly reflect on changes in consumer behavior, regulations, markets, and economic conditions, what is missed for overcoming social inequalities is the accompanied awareness or account for the impact of undertaking actions on society. As mindfulness induces general awareness, we argue that a collective ability that increases awareness about, for example, hidden customer needs do not cover the key dimension of the proposed new collective capability. What fundamentally differentiates collective social justice awareness from other reflective organizational practices is the consideration on how organizational course of actions jeopardize or alleviate social inequalities. It considers what social impact different oragnizational practices in response to changes in consumer behavior, regulations, markets, and economic conditions will have on the development of social justice in society. We argue that when mindful of social impact, organizations, in their usual business considerations, reflect on how the course of their action shape present and future development of society.

Following our conceptualization, collective social justice awareness will be maintained within organizations as long as individuals stay mindful and the processes and structures are implemented. When established processes and structures are radically changed, companies can lose the ability to recognize inequality and social justice issues ingrained in their practice.

## Discussion

Organizations now need to develop new organizational capabilities to overcome social inequalities embedded in their practices and, hence, to enhance their overall sustainable development (Harrison et al., 2019; Lazzarini, 2021). In addition to previous theories (Campbell, 2007; Carroll, 1979, 2021; Hunt, 2017; Maon et al., 2009; Wood, 1991), we proposed to apply the theory of mindfulness to explain how it can contribute to the development of sustainable organizations. As social inequalities are reinforced by automatic and habitual organizing steaming from the past (Amis et al., 2017, 2020), mindfulness, by increasing the present awareness of the organizational impact on society, helps to examine and question the correctness of taken-for-granted organizational practices crucial to ensure future sustainable development. From our perspective, it will lead to changes in habitual organizational practices that fuel social inequalities.

To sustain our proposition, in our review, guided by the microfoundational perspective of organizational capability (Felin et al., 2012), we conceptualize individuals, processes, and structures that, collectively, enable organizations to form MC for Social Justice. We perceive it as an organizational capability that reflects the extent to which an organization is aware of its impact through organizational practices on social justice in society. Notably, when developed in organizations, MC for Social Justice will lead to the development of a collective awareness of how various practices of hiring, decision-making, compensation, and role allocation impact the society organization operates. We argue that if an organization possesses a collective social justice awareness, it will be aware of the impact it may have on society as such, will be more prompt to intervene to correct not responsible habitual organizing. In line with the previous research that recognizes awareness as an essential and primary element for constituting responsible and sustainable organizing (Maon et al., 2009), we propose mindfulness as a useful approach to develop and instill higher awareness of social justice issues on both individual and collective levels what will boost reconsideration of non-socially sustainable organizational practices which frequently fuel social inequalities.

We contribute to the previous research that recognizes the importance of collective social justice perception (Schminke et al., 2015). With our study, we show how mindfulness may lead to the development of a collective perception of social justice in organizations or social justice awareness (as defined here). Moreover, by discussing organizational processes and structures, we provide evidence on what shared contextual factors may drive collective perceptions of social justice. Most importantly, as concerns the impact of organizational practices on society, the application of mindfulness in organizations may far exceed the perception of social justice within organizational boundaries and focus on broader issues of societal concerns.

In this vein, we also suggest mindfulness as an important characteristic of responsible leadership. Because responsible leadership requires the ability to bring people from different cultural backgrounds into teams, include different voices in dialogue, and reconcile intercultural and interpersonal dilemmas, among other things (Maak & Pless, 2006), being aware of the impact one’s action can have on others is an important characteristic. This is consistent with previous research stating that the awareness resulting from greater cognitive complexity enables leaders to discern interests, demands, and needs at different levels and levels (self, organization, and others in business and society) (Maak et al., 2016). Our conceptualization of Individual Social Justice Awareness may be of contribution to the literature on responsible leadership development.

Moreover, we argue that MC for Social Justice can enhance research in the field of mindful marketing (Sheth et al., 2011), where methods based on social justice awareness can steer marketing initiatives toward more sustainable and socially just. Notably, marketers can more effectively assess the impact of marketing practices (pricing, promotion, or product development) to make an informed decision that will bring positive societal results.

Moreover, as mindfulness in organizations is studied at both the individual and collective levels (Sutcliffe et al., 2016), we contribute to the literature in both directions. On an individual level, despite the vast body of knowledge about mindfulness, the research into the relationship between individual mindfulness and social justice is still in its infancy (Rashkova et al., 2022; Sajjad & Shahbaz, 2020; Wamsler et al., 2018) and results in a dispersed and fragmented body of knowledge (Hick & Furlotte, 2009; Schuh et al., 2019; Yu et al., 2020). We explained how mindfulness contributes to the development of greater sensitivity toward social justice. Notably, we provided evidence that, at the individual level, mindfulness translates into specific characteristics, namely, inner awareness, outer awareness, non-discriminatory awareness, present-oriented awareness, and ethical-minded awareness, that explain different behavioral, cognitive, and affective individual resources that comprehensively contribute to the development of social justice awareness and lead to more socially just behavior.

On a collective level, previous research has mostly studied mindfulness as a quality to discern individual attention to increase organizational resilience (Fraher et al., 2017; Sutcliffe et al., 2016; Vogus & Sutcliffe, 2012). By conceptualizing MC for Social Justice, we expand the purpose of mindfulness in organizations, suggesting that it can give rise to a new organizational capacity that can lead to a high collective awareness of social justice issues and encourages collective efforts to overcome social inequality.

Furthermore, previous research has frequently noticed a missing understanding of the connection between individual and collective mindfulness (Sutcliffe et al., 2016). Thus, by presenting a multi-level framework that combines different perspectives (individual and collective), we explain how individual-level mindfulness can lead to collective-level mindfulness. In the context of MC for Social Justice, mindfulness contributes to the individual awareness for social justice, which in turn elicits some collective organizational processes and structures occurring on a collective level.

By conceptualizing MC for Social Justice, we respond to numerous calls of previous research to broaden the implication of mindfulness to the societal level (e.g., Sajjad & Shahbaz, 2020; Wamsler et al., 2018). We provide some initial insights into how mindfulness can help organizations reframe their operations and practices with greater inclusion of social justice into the organizational agenda.

### Managerial Implications

Overcoming social inequalities represents a challenge for many organizations embracing sustainable development. Mindfulness, which has seen a strong interest in the managerial literature and practice, offers an interesting implementation in this regard. With our study, we propose mindfulness as a specific organizational capability that brings attention beyond the company’s operations and activities and enables companies to adopt a set of practices useful to account for social justice challenges. To assist managers in applying our theoretical framework, below, we suggest that at first, they should measure the level of collective social justice awareness and, secondly, if necessary, they can develop or extend its application in organizations.

We propose several ways organizations may understand the level of collective social justice awareness development. Since our review has shown that individuals represent an indispensable base point for developing collective social justice awareness, organizations should assess the level of leaders’ and employees' mindfulness taking into account organizational context. Moreover, since mindfulness is an ability that can be trained, the availability of mindfulness training in organizations will indicate the possibility of developing Mindfulness Capability for Social Justice. Finally, the presence of individualized processes and structures will also highlight the possible presence of collective awareness in organizations. As discussed earlier, the more organizational processes and structures are implemented, the more likely organizations will have a Mindfulness Capability for Social Justice.

As highlighted in the upper part of our study, Mindfulness Capability for Social Justice starts with individual mindfulness. As such, to develop collective social justice awareness, organizations need to allocate the resources to ensure the availability of mindfulness training and corresponding facilities. Moreover, HR managers may also include mindfulness in attitudinal measurement tests to ensure the presence of mindful employees in the workplace. Nevertheless, collective mindfulness is not about mindful employees working together, it is a dynamic process of mindful people and adopting mindful organizational practices. The adoption of suggested organizational processes (developing ethical practices for social justice, adopting social justice values, encouraging interconnectedness, and encouraging diverse perspectives and inclusivity) and two organizational structures (non-discriminatory organizational climate and authentic and ethical leadership) in conjunction with the availability of mindful employees will lead to the development of Mindfulness Capability for Social Justice.

With this study, we encourage practitioners to develop Mindfulness Capability for Social Justice, which is important for both organizations and societies. From a societal perspective, organizations, as having the resources to shape the development of our societies, when aware of social justice issues, develop initiatives, products, and solutions that alter human development. From an organizational perspective, business leaders and organizations with high social justice awareness are more likely than competitors to be aware of and respond more rapidly to stakeholder concerns. By arriving at balanced decisions that form sound policies, managers will be more inclined to build support systems that sustain the development of a socially prosperous society. In this turn, contribution to the development of a just and prosperous society will undoubtedly positively impact investors' attractiveness and customer loyalty and, all in all, facilitate business growth. Thus, there is a mutual benefit to the development of a higher collective social justice awareness in organizations since the health of business and society is strictly dependent (Drucker, 1969).

### Limitations and Future Research

We recognize that this study has some limitations. While presenting a conceptual view of the MC for Social Justice, this framework is not empirically validated. We encourage future research to develop a scale for Mindfulness Capability for Social Justice and empirically test the proposed framework to enrich the relationship between individualized components. Moreover, we have analyzed a specific direction of the relationship: from the individual ability to collective capability. Future research should explore the opposite direction: from the collective to the individual to understand how the mindfulness implemented in organizations affects the behavior of employees both within and outside the organization.

In our research, we emphasize that the sense of connectedness within organizations is a fundamental process. However, the way people interact and connect has changed after the Covid pandemic (Kumar et al., 2020), leading to the wider adoption of digital solutions. As such, future studies could try to understand the impact of digital tools on MC for Social Justice.

Furthermore, we conceptualize the organizational capability for social justice as a collective construct, which suggests variance within organizations over time and, at a given time, variance across organizations. We join the call of several scholars (Donald et al., 2019; Reina & Kudesia, 2020; Wan et al., 2020; Wang et al., 2021) to explore the organizational context, which we suggest plays a role in developing MC for Social Justice.

While our research yields some important results, we recognize that introducing mindfulness within organizations alone will not ensure human progress toward a socially just society. Joint efforts are required. With our study, we encourage scholars from different fields to explore how the concept of mindfulness can be implemented in other fields to achieve positive results in society.

## Data Availability

All data generated or analysed during this study are included in this published article.

## Footnotes

1 The aforementioned perspective belongs to the Western approach. The Eastern approach, on the other hand, comes from meditative practices and is characterized by moment-to-moment, nonjudgmental awareness that is cultivated in the present moment (Kabat-Zinn, 2005; Shapiro et al., 2006). For a detailed overview of the difference between the two, see Hart et al. (2013).
